# Neurodevelopment in perinatally HIV-infected children: a concern for adolescence

**DOI:** 10.7448/IAS.16.1.18603

**Published:** 2013-06-18

**Authors:** Barbara Laughton, Morna Cornell, Michael Boivin, Annelies Van Rie

**Affiliations:** 1 Children's Infectious Diseases Clinical Research Unit, Department of Paediatrics and Child Health, Stellenbosch University, Cape Town, South Africa; 2 Centre for Infectious Disease Epidemiology & Research, School of Public Health & Family Medicine, University of Cape Town, Cape Town, South Africa; 3 Departments of Psychiatry and Neurology/Ophthalmology, Michigan State University, East Lansing, MI, USA; 4 Department of Epidemiology, University of North Carolina at Chapel Hill, Chapel Hill, NC, USA

**Keywords:** adolescents, children, perinatally HIV infected, neurodevelopment, neurocognitive, neurological, hearing, executive function

## Abstract

Globally, an estimated 3.4 million children are living with HIV, yet little is known about the effects of HIV and antiretroviral treatment (ART) on the developing brain, and the neurodevelopmental and behavioural outcomes of perinatally HIV-infected (PHIV+) adolescents.

We reviewed the literature on neurodevelopmental outcomes in PHIV+ children and adolescents, and summarized the current evidence on behaviour, general cognition, specific domains, hearing and language, school performance and physical disabilities due to neurological problems.

Evidence suggests that PHIV+ children do not perform as well as controls on general cognitive tests, processing speed and visual–spatial tasks, and are at much higher risk for psychiatric and mental health problems. Children with AIDS-defining diagnoses are particularly at risk for poorer outcomes.

A striking finding is the lack of published data specific to the adolescent age group (10–25 years), particularly from resource-constrained countries, which have the highest HIV prevalence. In addition, extreme heterogeneity in terms of timing and source of infection, and antiretroviral experience limits our ability to summarize findings of studies and generalize results to other settings.

Due to the complex nature of the developing adolescent brain, environmental influences and variation in access to ART, there is an urgent need for research on the longitudinal trajectory of neurodevelopment among children and adolescents perinatally infected with HIV, especially in high burden resource-constrained settings.

## Introduction

An estimated 3.4 million children are living with HIV worldwide [1], 28% of whom have started antiretroviral therapy (ART) [2]. Yet, little is known about the effects of HIV and ART on the developing brain and the neurodevelopmental outcomes of perinatally HIV-infected (PHIV+) adolescents.

In neuropsychological terms, adolescence spans the age range of 10–25 years [3], which in 2013 includes those born between 1988 and 2003. Over this time period, the management of PHIV+ infants, children and adolescents changed dramatically. Before the introduction of ART in 1995, 50% of PHIV+ children died before the age of two [4], with a few slow progressors surviving to adolescence [5]. Prior to 1997, children in Europe and the United States may have received multiple antiretroviral regimens, including those that would now be considered suboptimal therapy. In 1997, combination ART was introduced in the United States. Since 2004, access to ART has expanded rapidly in resource-poor settings and depending on the country, 28–80% of treatment-eligible children have initiated ART [2].

Context-specific differences in access to ART over the past two decades have resulted in great variability in disease severity and in exposure to ART among PHIV+ adolescents: some started ART soon after HIV infection, prior to clinical diagnosis of neurodevelopmental delay [6]; some initiated ART after the diagnosis of HIV encephalopathy [7, 8], resulting in neurological deficits that remained permanent despite ART [9, 10]; other PHIV+ adolescents are slow progressors and remain ART-naïve as they have not yet reached the ART eligibility threshold [2]. The source and time of infection cannot always be determined in HIV+ adolescents, especially in settings with generalized HIV epidemics.

There is also substantial heterogeneity in the literature in terms of the age of study participants. Most published studies have focused on younger children aged 6–12 years [11–13] or 7–16 years [14], crossing from childhood to adolescence. The issue is further compounded by the measures used to assess functionally relevant outcomes for PHIV+ adolescents in diverse cultural settings. In some settings, the emphasis may be on achieving good school grades to maximize employment options, while in other settings adolescents may be more concerned about starting a family and providing resources to support a household or extended family.

Given this extreme heterogeneity in the age of study participants, severity of disease, antiretroviral experience, and the definition and measurement of outcomes, we reviewed the literature on neurodevelopmental outcomes in PHIV+ adolescents.

We conducted a literature search using the following key words: neurodevelopment/al, development, neurocognitive, cognitive, adolescents, youth, perinatal/vertical HIV-infected, HIV exposed, school performance, adaptive functioning, hearing and neuroimaging. We reviewed bibliographies and relevant articles from different contexts globally, limited to the most recent papers. We included all ages spanning adolescence. The original aim was to review evidence on neurodevelopmental outcomes among perinatally HIV-infected adolescents. However, the paucity of strictly adolescent data was striking. In the absence of these data, we drew on published studies of neurodevelopmental in younger children with HIV as a guide to what could be expected to impair neurodevelopment in adolescence.

## Neurodevelopmental changes during adolescence

The key developmental tasks during adolescence are to develop an identity, to become more independent, and to consider the future in terms of career, relationships, families, housing, etc. [15]. Traditionally, adolescence is viewed as the age when abstract thought develops, together with improvements in memory, language, processing speed, attention and concentration [16]. A more contemporary view is that the major dimensions of cognitive development during adolescence are the refinement of executive control and the attainment of a more conscious, self-directed and self-regulating mind [17–19]. Central to these are executive function (EF) processes such as voluntary response inhibition, working memory, response planning, improved processing speed, cognitive flexibility, and rule-guided behaviour [18, 19].

While the adolescent's brain does not increase substantially in volume, changes in maturation reflect reorganization of regulatory systems and correlate with neurocognitive and behavioural outcomes [17]. During adolescence, white matter increases in a linear fashion with increased myelination and re-organization with synaptogenesis and pruning, especially in the frontal lobes and prefrontal cortex, which serve as the governor of cognition and action [17, 18]. Maturational changes are influenced by numerous factors including genetic and environmental factors as well as overall health status, resulting in variation between children and within the same child for the various domains of neurodevelopment. The impulsive and risk-taking behaviour of adolescents is also thought to be a consequence of the interaction of social context and the development of judgment, decision-making and internal control [17, 20].

Neuropathology caused by HIV is most evident in basal ganglia and cerebral white matter. Neuronal loss is prominent in the prefrontal cortical regions, which may cause difficulty in complex mental processing [21]. These are the regions where myelination and remodelling of synaptic connections are still occurring during adolescence [22, 23]. When coupled with the high risk for psychiatric difficulties in PHIV+ adolescents, the relationship between impaired EF and risk-taking behaviour can be compounded.

## General cognition

The most common measure of neurodevelopmental outcome is general cognition. General cognitive assessments provide a global score of performances in various domains. In this case, appropriate perinatally HIV-unexposed (PHU) children can be used as controls [12]. Neighbourhood-matched perinatally HIV-exposed uninfected (PHEU) children can be used to control for confounding effects of prenatal HIV exposure, ART exposure and maternal illness, etc. [11, 24], but they should not be seen as an ideal control group.

Table 1 summarizes recent studies of general cognition in PHIV+ children. Most neurocognitive assessment studies of PHIV+ children have been performed in the United States and Europe [14, 25–27], though some studies from other continents have been published [11–13, 24]. There are many differences between the study populations, with each group having particular areas of vulnerability of the brain and life experiences (e.g., higher drug abuse and lower adherence in some parts of the United States; higher poverty and lower access to comprehensive treatment in some limited-resource countries; different treatment when infants). Overall, PHIV+ children and adolescents perform more poorly in neurodevelopmental assessments than PHU controls or national norms [11, 13, 24], although in some studies there were no significant differences between groups [12, 26, 27]. For children in the United States, better cognitive outcomes have been associated with having a biological parent as caregiver, higher family income level, and higher caregiver cognitive function [14]. In PHIV+ children with less severe disease progression (WHO clinical stage I or II) and those on ART without a history of an AIDS defining illness, overall cognitive development has been found to be similar to that of PHEU children [14] although still significantly poorer than PHU children [13].

**Table 1:** Summary of recent studies on general cognition in HIV-infected children

Study	Participants	Age (range)	Measure	Findings	Antiretroviral therapy
Koekkoek *et al*. 2008 [26] The Netherlands	22 PHIV+	Median 9.46 years (6–13.5)	SON-R	No gross cognitive deficits compared to normative values	Median age HAART initiation 5.6 years
Smith *et al*. 2012 USA+Puerto Rico [14]	270 PHIV+/noC 88 PHIV+/C 200 PHEU	7–16 years	WISC-IV	Scores significantly lower for PHIV+/C group afteradjusting for covariates 77.8 vs. 83.4 and 83.3	Median age: first ART 0.6 years; first dual therapy 1.25 years
Blanchette *et al*. 2002 [27] Canada	14 PHIV+ 11 control siblings	6.3–14 years	WISC-R or WISC-III	Mean FISQ 91.7 vs. 100.5	12 were on ART
Ruel *et al*. 2011 Uganda [13]	93 PHIV+ 106 HIV−	Median 8.7 years (6–12)	KABC-2	PHIV+ performed worse than HIV- children	All children above WHO threshold for ART initiation
Bagenda 2006 Uganda [12]	28 HIV+ 42 HEU 37 HIV−	6–12 years	KABC	No significant difference	ART-naïve
Wood 2009 USA [25]	81 PHIV+ 38 PHIV+/C 43 PHIV+/noC	Median 15.2 years (11–24)	WISC-IV or WASI	Median FISQ of PHIV+/noC fell within normal range; Median FISQ of HIV+/C in below average range	Median age: ART initiation: 3.1 years; Median age: HAART initiation: 6.5 years
Puthanakit *et al*. 2010 [11] Thailand	39 HIV + 40 – affected 42 healthy controls	Median 9.3 years (6–12)	WISC-III	Mean FISQ of HIV+ and affected groups significantly lower than healthy controls 79 vs. 88 vs. 96 *p*<0.01	87% on ART for median of 35 weeks (IQR 29–53)
Puthanakit *et al*. 2013 [29] Thailand, Cambodia	284 PHIV+, 155 PHEU, 164 PHU	Median age 9 years (1–12)	WISC-Thai	No difference between early and deferred ART initiation RCT arms. PHIV+ children performance worse than PHEU and PHU on IQ	Early versus deferred HAART at enrolment from 1 to 12 years of age
Hoare *et al*. 2012 South Africa [24]	12 PHIV+ 12 HIV – community controls	8–12 years	WASI: verbal performance	Mean scores: 87.8 vs. 101.2 73.7 vs. 85.7	ART-naïve

PHIV+/C Perinatally HIV-infected with a previous class C event. PHIV+/noC Perinatally HIV-infected with no past history of class C event. KABC Kaufman Assessment Battery for children. KABC-2 Kaufman Assessment Battery for children, 2nd edition. SON-R Snijders-Oomen nonverbal intelligence test for children and adolescents (abridged). WASI Wechsler Abbreviated Scale of Intelligence. WISC-R Wechsler Intelligence Scale for Children –Revised. WISC-III & IV Wechsler Intelligence Scale for Children versions 3, 4. WISC-Thai Wechsler Intelligence Scale for Children Thai version.

Martin and colleagues evaluated predictors of cognitive decline in older children in the United States who had been on ART for at least a year. Overall, PHIV+ children on ART remain at risk for developing CNS disease, with children with minimal to moderate CT brain scan abnormalities scoring significantly lower than children with normal scans on composite measures of cognitive ability [21]. The risk in asymptomatic adolescents was confirmed in a small pilot study which found a higher rate of neurocognitive impairment in asymptomatic adolescents compared to adults >60 years old (67 vs. 19%) [28].

Few studies have addressed the effect of ART initiation on cognitive development in PHIV+ school-age children and adolescents in low- and middle-income countries. In a cohort of Thai children, cognitive function did not improve in response to ART, even in children who achieved virological suppression and immunological recovery [11]. There were also similar neurodevelopmental and neuropsychological outcomes in Thai and Cambodian children between early and deferred ART groups, although both groups performed worse than PHEU children [29]. A small study of younger South African children (median age five years) also failed to observe neurodevelopmental improvement following ART initiation [9].

## Specific domains of cognitive development

Global cognitive scores may overlook subtle deficits in one or more areas specific to PHIV+ children and may affect their performance on a different level [26, 30, 31]. For example, even in PHIV+ children with global cognitive scores in the normal range, EF may be impaired, especially in children with cortical atrophy, lower fractional anisotropy of the corpus callosum and those with CD4+ counts below 500 cells/mm^3^ [21, 24].

Specific domains may be measured as subtests on cognitive assessments or by a test specifically designed for that purpose. The development of EF starts in childhood, but is highly important in the development of adolescents. EF is a composite of different domains including processing speed, response inhibition, working memory, response planning, cognitive flexibility with task switching, attention and concentration [32]. Processing speed is associated with increased capacity for working memory, enhanced inductive reasoning and greater accuracy in solving arithmetic word problems, and consistently predicts performance on cognitive tasks [16].

Table 2 summarizes studies that explore the impact of HIV on important neurocognitive domains. PHIV+ children have been found to perform significantly poorer in EF tasks, particularly in terms of processing speed [13, 14, 26, 33], memory [12, 14, 21, 24, 34] and attention [13]. Lower scores on visual–spatial processing have also been described in younger PHIV+ children [27, 35]. Visual–spatial processing is important for adolescents as it impacts on reading, writing and learning. PHIV+ children have been shown to be slower and less accurate on pattern recognition [26], and to have lower scores than controls on sequential processing, simultaneous processing [36], planning/reasoning [13] and visual memory [24].

**Table 2:** Specific neurocognitive domains affected in perinatally HIV-infected children

Study	Participants	Age (range)	Measure	Findings
*Processing speed:*				
Koekkoek *et al*. 2008 [26] The Netherlands	22 PHIV+	Median 9.5 yrs (6–13.5)	Amsterdam neuro-psychological task: baseline speed	Significantly slower compared to age-appropriate norms
Smith *et al*. 2012 [14] USA+ Puerto Rico	88 PHIV+/C 270 PHIV+/NoC 200 PHEU	7–16 years	WISC-IV Processing speed	Lower scores on processing speed for PHIV+/C compared to PHIV+/NoC and PHEU. PHIV+/NoC and PHEU scores were similar
Nachman *et al*. 2012 [34] USA	319 PHIV+ IQ>70	6–17 years	WISC-IV coding recall	Higher peak viral load (>100 000 copies/ml)and lower nadirCD4% (<15%) associated with slower speed
Ruel *et al*. 2012 Uganda [13]	93 PHIV+ 106 PHU CD4≥15% CD4 count ≥350 cells/µl	Median 8.7 yrs (6–12)	Test of variables of attention	Worse visual, auditory and overall reaction time than HIV-community age matched
*Set Shifting:*				
Koekkoek *et al*. 2008 [26] The Netherlands	22 PHIV+	Median 9.5 yrs (6–13.5)	Amsterdam Neuro-psychological task: Attentional flexibility	Significantly slower compared to age-appropriate norms Better outcomes with longer HAART duration
*Verbal Fluency: (EF in the verbal domain)*				
Koekkoek *et al*. 2008 [26] The Netherlands	22 PHIV+	Median 9.5 yrs (6–13.5)	Verbal fluency	Significantly lower scores compared to age appropriate norms
Hoare *et al*. 2012 [24] South Africa	12 PHIV+ 12 HIV−	Mean 10.4 yrs (8–12)	Semantic fluency	Significantly lower than HIV-negative controls from same neighbourhood
*Memory*				
Blanchette *et al*. 2002 [27] Canada	14 PHIV+ 11 control siblings	6.3–14.9 yrs	WISC-digit span and information Story recall Rey Complex figure	No difference between groups
Bagenda 2006 Uganda [12]	28 HIV+ 42 HEU 37 HIV−	6–12 years	KABC Sequential processing (Immediate memory recall)	HIV+ significantly lower scores than HEU No difference between PHIV+ and HIV- groups
Martin *et al*. 2006 [21] USA	41 PHIV+	Mean 11.2 yrs (6–16)	WISC III – working memory: Digit span backwards Arithmetic	Significantly lower scores in those with abnormal CT brain scans compared to those with normal scans
Hoare *et al*. 2012 [24] South Africa	12 PHIV+ 12 HIV−	Mean 10.4 yrs (8–12)	Working memory: WISC IV digit span Backward Visual memory: Rey complex figure	Groups performed similar for working memory Visual memory significantly worse in PHIV+ compared to HIV- controls
Smith *et al*. 2012 USA+Puerto Rico [14]	270 PHIV+/noC 88 PHIV+/C 200 PHEU	7–16 years	WISC IV: Working memory	2 to 5 fold increased risk of impairment for HIV+/C group compared to PHEU group
*Visual spatial memory/integration*				
Koekkoek *et al*. 2008 [26] The Netherlands	22 PHIV+	Median 9.5 yrs (6–13.5)	Amsterdam neuro-psychological task: visuospatial memory	Significantly lower scores in visuospatial working memory compared to age-appropriate norms.
Puthanakit *et al*. 2013 [29] Thailand, Cambodia	284 PHIV+, 155 PHEU, 164 PHU	Median 9 yrs (1–12),	Beery Visual Motor Integration	No difference between early and deferred ART initiation RCT arms PHIV+ children performance worse than PHEU and PHU
Hoare *et al*. 2012 [24] South Africa	12 PHIV+ 12 HIV−	Mean 10.4 yrs (8–12)	Spatial processing: WASI block design Rey complex figure test	Significantly worse than HIV-negative controls

PHIV+/C: Perinatally HIV-infected with a previous class C event. PHIV+/noC: Perinatally HIV-infected with no past history of class C event.

## Adaptive functioning

Adaptive functioning has been defined as the ability to function effectively in a number of settings requiring social and problem solving skills, including school, home and social settings [37]. Cognitive assessments may not be the appropriate measurement tools to capture the ability of children and adolescents to function in real life situations. For example, in child-headed households in resource-constrained settings, children are required to take far more responsibility than in resource-rich countries. Measuring adaptive functioning, as previously used in younger children, may provide a more meaningful way of assessing how adolescents are functioning in their own environments. There is conflicting evidence on the correlation of scores. Gosling *et al*. found significant weakness in adaptive functioning compared with cognitive functioning in PHIV+ children [38]. In contrast, Smith *et al*. found some disparity, with higher scores in adaptive function at lower cognitive scores [14]. As the number of PHIV+ children grow, further research on this is needed to determine whether measuring adaptive functioning is a useful measurement tool for neurodevelopmental outcomes in PHIV+ adolescents in less developed settings.

## The interplay between HIV, neurodevelopment, behaviour and mental health

Several studies have focused on the burden of psychiatric problems and mental health functioning impairment in PHIV+ children and the interplay with EF, risk-taking behaviour and treatment adherence.

A study in the United States observed a 25% prevalence of mental health problems among PHIV+ children and adolescents, well above that of the general population though lower than the 38% rate observed in the PHEU comparison group [39]. Caregiver characteristics (psychiatric disorder, limit-setting problems and health-related functional limitations) and child characteristics (younger age and lower IQ) were most predictive of the occurrence of mental health problems. Another US study documented that 18% of 6–17 year old PHIV+ children had a lifetime history of psychiatric medications, 13% were on medication (largely stimulants and antidepressants) for psychiatric problems and 22% had a past or current history of non-medication psychological intervention [40].

There is a strong association between psychological and neurocognitive functioning. In a study in Atlanta and New York City, depressive symptoms in PHIV+ adolescents were best predicted by a combination of negative coping skills and poor neuropsychological functioning. Conduct disorder problems were directly associated with neuropsychological functioning (cognitive inflexibility) and negative coping skills [41]. A study in New Zealand reported that risky personality and performance on the neuropsychological and EF tests were significant predictors of risk-taking [42]. Furthermore, psychiatric disorders and behavioural health challenges in PHIV+ children can lead to poor ART adherence, risk-taking behaviour, including risky sexual behaviour, precocious sexual debut, teenage pregnancy and substance abuse [40, 43–48].

These findings add weight to the increasing concern about long-term neurodevelopmental problems among PHIV+ adolescents [8, 49] and the burden that these pose for individuals, families and the education and health care systems.

## Language and hearing

As children transition to early and middle adolescence, language and reading skills are the critical building blocks for literacy and future academic success, with an important transition from “learning to read and reading to learn” [50]. There is evidence that verbal skills are negatively affected in PHIV+ children [14, 24, 36, 50, 51]. In a large study in New York City, vocabulary and reading were worse in PHIV+ youths compared to PHEU, even after adjusting for demographic variables [50]. In contrast, Rice *et al*. in a multisite US (including Puerto Rico) study found that both PHIV+ and PHEU performed poorly on verbal tests, but there was no difference between the two groups [51].

While CD4 cell count, HIV viral load and CDC Classification were not associated with verbal scores in the New York City study [50], two other US studies found that a history of an AIDS-defining illness was associated with verbal comprehension impairment [14, 51]. In addition, Rice *et al*. found that after controlling for cognitive and hearing impairment, children who were PHIV+ with detectable viral load and ART initiation less than six months of age had an increased risk of language impairment. Other risk factors for language impairment combined with cognitive or hearing impairment were race/ethnicity, caregiver's education and intelligence quotient (IQ) status and having a non-biological parent as caregiver [51].

Adjustment for hearing deficits in language assessment of PHIV+ children is important as the prevalence of hearing loss in PHIV+ children is high and ranges from 20% in higher income countries to 38% in low-resource settings [52, 53]. In resource-poor settings, hearing loss was largely conductive, including chronic suppurative otitis media and dry tympanic membrane perforations, which may reflect the lack of consistent otological care, whereas in well-resourced settings more children had sensorineural hearing loss [54], which may possibly be related to measurement in the United States. A low CD4 count and a history of AIDS-defining illness were associated with both hearing and language impairment [53, 54].

## School performance

School performance is a functional outcome that is highly relevant in terms of future quality of life and employment prospects [55]. Academic failure predicts problems in schooling and leads to an increase in school dropouts [56]. Insight into the school performance of PHIV+ children is important in order to plan appropriate resources to support this vulnerable population. However, accurate measurement is problematic due to the abundance of potential confounders. A child's school performance is dependent on numerous variables including social and family factors [55, 56]. In addition, the indirect effects of HIV infection including hearing loss, school absenteeism due to ill health or ART management, depression and/or social problems need to be considered when interpreting school performance [33].

Several studies have explored school performance among PHIV+ children and adolescents, and identified poorer outcomes compared with children without HIV, with the exception of a French study, which reported an academic failure rate of 16%, similar to the general population [57]. Outcome measurements were highly variable and included 42% with a learning disability [25], 27–33% receiving special education [50, 57, 58], 15% having repeated two or more grades [57] and 51% having failed at least one grade [59]. Limited caregiver education or intelligence level increased the risk of poor educational outcomes [14]. There is a striking lack of studies on academic achievement in resource-limited countries. Although such research would be difficult to undertake, it would provide valuable information to guide interventions.

## Physical disabilities due to neurological problems

Physical problems due to HIV encephalopathy have been well described in the pre-ART era [10]. There is however a paucity of data on neurological outcomes of ART-naïve PHIV+ child non-progressors as well as those on ART, particularly in older children. In Uganda, Bagenda *et al*. describe children with hypotonia, hyperreflexia and delayed milestones, which disappeared as they grew older [12]. Boivin *et al*. also found motor impairment in PHIV+ asymptomatic children in the first two years of life and later in childhood (ages 8–12 years) [36].

Two South African studies described motor deficits and neurological manifestations in PHIV+ children [9, 60]. Govender *et al*. reported 59% abnormal neurological examinations in children aged one month to 12 years, 41% with global pyramidal long tract signs and 16% with cortical visual impairment. However, there were many participants with neurological sequelae due to secondary infections and the direct effects of HIV infection are not clear [60]. Smith *et al*. found evidence of motor dysfunction in 33% of ART-naïve children with no improvement after six months of treatment [9]. In a cohort of 210 PHIV+ French children followed since birth, at a median age of 15 years, three children had persistent motor dysfunction and five had mild to moderate physical impairment, indicating a low incidence of physical disabilities due to neurological problems in children who gain timely access to ART [57]. Some of the neurological manifestations in the young child may not be reversible and may still be evident in the adolescent. We have included these studies to emphasize that a neurological examination should be included when measuring functional outcomes in PHIV+ adolescents.

CNS disease and stroke have been documented as causes of death in PHIV+ children, adolescents and young adults in the USA [8]. In the pre-ART era, the annual risk of cerebrovascular events was 1.3% [61], but there are no data on the incidence of stroke in PHIV+ children on ART. Similarly, the incidence and effect of central nervous system insults caused by infections such as tuberculosis and meningitis have not been well documented.

## Markers of HIV disease progression and severity

Traditional markers of HIV disease progression and severity including high plasma viral load, lower CD4 cell counts and/or CD4%, and history of an AIDS defining illness have been associated with poorer neurocognitive performance [13, 14, 21]. In addition, some markers of vascular dysfunction and T-cell activation have been found to correlate with global cognitive outcomes in PHIV+ youth. Specifically, higher soluble P-selectin, a marker and mediator for inflammatory vascular disease, and lower fibrinogen (a pro-coagulant state marker) have been associated with poorer cognitive function [62]. CD4^+^ activation and, under certain circumstances, CD8^+^ activation have been shown to have favourable neurodevelopmental implications in PHIV-infected children [63]. In a study of ART-naive Ugandan children, HIV subtype-A was associated with higher viral loads and poorer performance compared to subtype-D, suggesting that subtype-A may be more neuropathogenic in children [64].

## Intervention strategies

Studies have found that ART alone is not sufficient to reverse the neurodevelopmental consequences of HIV infection [31, 65]. Highly active ART (HAART) may even contribute to neuromotor decline over time [31, 66]. The inability of ART alone to restore HIV+ children to “normal” neuropsychological performance is a compelling rationale to evaluate alternative interventions for neurocognitive disability in paediatric HIV. Despite knowledge of deficits in PHIV+ children, there have been very few intervention studies. One intervention is computerized cognitive rehabilitation training [67, 68]. Preliminary results in Uganda indicate that using computer games for cognitive rehabilitation can be of great benefit to PHIV+ children and adolescents [69]. For younger children with HIV, caregiver training on practical strategies to enrich the developmental milieu of these children can also have significant neurocognitive benefit [70].

There is evidence to suggest a strong link between psychological well-being and the immunological impact of disease progression [71]. HIV-infected children who exhibited signs of resilience tended to have better neurodevelopmental functioning, social–emotional and gross motor functioning [72]. Some approaches to fostering resilience in PHIV+ children have centred around family dynamics within a cultural framework [73–76]. In a qualitative study of resilience among Rwandan HIV-affected children and families [74], Betancourt and colleagues identified five factors that increased resilience in children and families affected by HIV: perseverance, self-esteem/self-confidence, family unity/trust, good parenting, and collective/communal support. Interventions and strategies to leverage these resources may help to prevent mental health problems in these children as they grow into adolescence and adulthood [73]. Psychosocial intervention may also significantly enhance subsequent neurocognitive development of the child in response to the direct physiological, psychological, social and immunological impacts of this disease. For example, Coscia *et al*. showed that home environment had a stronger association with child IQ during the advanced than the early stages of disease [77]. Parental support has been shown to provide a stress-buffering effect for the effects of depression in these school children, that seemed to improve psychosocial and cognitive development [78, 79].

## Discussion

Each child has their own set of unique factors that shapes their development, making it difficult to identify the relative contribution of different factors impacting on the neurodevelopmental outcomes of PHIV+ adolescents. While HIV has a direct effect on neurocognitive development, the effects of deprivation and poverty, quality of home environment, genetics, opportunistic infections, and access to care may overshadow the effects of HIV, particularly in resource-constrained settings.

Important variables that have been shown to affect neurodevelopmental outcomes include caregiver mental health or substance problems [14], orphan status and chronic illness [60], nutritional status [60, 80] formal education and home environment [80] as well as having a biological parent as caregiver, higher family income level and higher caregiver cognitive functioning [14]. Given the psychosocial impact of diagnosis and treatment, as well as the contribution of coping with cognitive weaknesses, additional attention to behavioural and mood symptoms associated with childhood HIV is essential.

It is possible that ART initiation in school-aged children and adolescents may be too late to reverse impairment. Cohorts initiating ART earlier report better outcomes, suggesting that earlier ART initiation is beneficial [6, 29]. However, there is inadequate evidence of the effects of long-term ART on the developing brain. Lower nadir CD4 counts, higher viral loads and the history of an AIDS-defining illness are associated with poorer neurodevelopmental outcomes, further supporting the need for early ART initiation in children. Children presenting with these risk factors should be offered neurodevelopmental screening as part of routine HIV care and referral to supportive services or formal assessments where appropriate. PHIV+ adolescents should be provided with multidisciplinary support services including adherence support, reproductive health counselling and mental health and educational/vocational planning [81]. Preliminary evaluations of these multi-faceted interventions for PHIV+ adolescents have shown good results in improving adherence and reducing risk-taking behaviours [81–84].

While most studies describe the proportions of male and female study participants, generally the data were not analysed and compared for sex differences in outcomes. This is possibly because it is generally accepted that the neurodevelopment of boys and girls are similar. However, possible sex differences in adolescent neurodevelopmental outcomes require further exploration.

## Conclusions

PHIV+ adolescents constitute a large heterogeneous population. Overall, HIV+ children and adolescents have poorer neurodevelopmental outcomes than uninfected peers, particularly those with more advanced HIV disease. There is also emerging evidence that PHIV+ adolescents are especially at risk for poorer psychiatric outcomes and EFs. However, the impact of HIV on the developing adolescent brain is highly complex, influenced by many factors and not well understood. Compounding and contributory factors may include an increased risk of substance use, risky sexual and other risk-taking behaviours, and poorer ART adherence.

A striking finding is the paucity of data specific to the adolescent age group (10–25 years) and the lack of longitudinal cohort studies designed to assess the effect of HIV on neurocognitive functioning in PHIV+ adolescents. While much of the current evidence is from younger ages, evidence from these studies provides valuable information as neurodevelopmental problems occurring at younger ages are likely to persist in adolescence and adulthood. Furthermore, the majority of studies on neurodevelopmental outcomes in adolescents are from the United States and Europe, with few studies from low- and middle-income countries which have the highest prevalence of PHIV+ adolescents. Few studies explore possible gender differences in adolescent neurodevelopment. Finally, little is known about the complex nature of recovery of the brain after initiation of ART. Thus, there is an urgent need for longitudinal research assessing the long-term effect of ART and timing of ART initiation on neurodevelopmental outcomes of perinatally HIV-infected adolescents by gender, particularly in resource-constrained settings.
